# Post-Injury Treatment with 7,8-Dihydroxyflavone, a TrkB Receptor Agonist, Protects against Experimental Traumatic Brain Injury via PI3K/Akt Signaling

**DOI:** 10.1371/journal.pone.0113397

**Published:** 2014-11-21

**Authors:** Chun-Hu Wu, Tai-Ho Hung, Chien-Cheng Chen, Chia-Hua Ke, Chun-Yen Lee, Pei-Yi Wang, Szu-Fu Chen

**Affiliations:** 1 Departments of Physiology and Biophysics, National Defense Medical Center, Taipei, Taiwan, Republic of China; 2 Graduate Institute of Life Sciences, National Defense Medical Center, Taipei, Taiwan, Republic of China; 3 Department of Obstetrics and Gynecology, Chang Gung Memorial Hospital at Taipei and College of Medicine, Chang Gung University, Taipei, Taiwan, Republic of China; 4 Department of Physical Medicine and Rehabilitation, Cheng Hsin General Hospital, Taipei, Taiwan, Republic of China; Nathan Kline Institute and New York University Langone Medical Center, United States of America

## Abstract

Tropomyosin-related kinase B (TrkB) signaling is critical for promoting neuronal survival following brain damage. The present study investigated the effects and underlying mechanisms of TrkB activation by the TrkB agonist 7,8-dihydroxyflavone (7,8-DHF) on traumatic brain injury (TBI). Mice subjected to controlled cortical impact received intraperitoneal 7,8-DHF or vehicle injection 10 min post-injury and subsequently daily for 3 days. Behavioral studies, histology analysis and brain water content assessment were performed. Levels of TrkB signaling-related molecules and apoptosis-related proteins were analyzed. The protective effect of 7,8-DHF was also investigated in primary neurons subjected to stretch injury. Treatment with 20 mg/kg 7,8-DHF attenuated functional deficits and brain damage up to post-injury day 28. 7,8-DHF also reduced brain edema, neuronal death, and apoptosis at day 4. These changes were accompanied by a significant decrease in cleaved caspase-3 and increase in Bcl-2/Bax ratio. 7,8-DHF enhanced phosphorylation of TrkB, Akt (Ser473/Thr308), and Bad at day 4, but had no effect on Erk 1/2 phosphorylation. Moreover, 7,8-DHF increased brain-derived neurotrophic factor levels and promoted cAMP response element-binding protein (CREB) activation. This beneficial effect was attenuated by inhibition of TrkB or PI3K/Akt. 7,8-DHF also promoted survival and reduced apoptosis in cortical neurons subjected to stretch injury. Remarkably, delayed administration of 7,8-DHF at 3 h post-injury reduced brain tissue damage. Our study demonstrates that activation of TrkB signaling by 7,8-DHF protects against TBI via the PI3K/Akt but not Erk pathway, and this protective effect may be amplified via the PI3K/Akt-CREB cascades.

## Introduction

Traumatic brain injury (TBI) triggers a complex cascade of apoptotic events which can contribute to delayed secondary injury processes [1]. Clinically, activation of caspases [2], profiles of Bcl-2 family proteins, and the release of pro-apoptotic proteins from mitochondria have been linked to TBI outcomes [3]. Experimental studies have also demonstrated that caspase inhibitors [2] or over-expression of Bcl-2, an anti-apoptotic molecule, were protective against TBI [4]. These studies indicate that apoptosis could serve as a therapeutic target following TBI.

On the other hand, TBI also activates endogenous protective mechanisms to counteract secondary injury [5]. Tropomyosin-related kinase B (TrkB) signaling has been considered to be an important protective mechanism induced by brain damage and a key regulator of neuronal survival [5], [6]. The TrkB is activated by binding to brain-derived neurotrophic factor (BDNF), which results in activation of downstream phosphatidylinositol 3-kinase (PI3K)/Akt, mitogen-activated protein kinase (MAPK)/Erk, or PLC-γ signaling via receptor autophosphorylation and dimerization [6], [7]. PI3K/Akt and Erk signaling pathways are the major TrkB-mediated survival pathways that promote neuronal survival and protect against apoptosis [6], [7]. In addition, BDNF/TrkB signaling can promote further BDNF production through cAMP-response element binding protein (CREB), a key transcription factor for BDNF induction via activation of PI3K/Akt or Erk signaling [8]–[10], which is emerging as a positive-feedback loop. TBI induces an acute but transient increase in BDNF and TrkB mRNA, presumably indicating a transient but failed endogenous protective response [11], [12]. These data suggest that modulation of TrkB signaling have a therapeutic role in brain damage. However, recombinant BDNF has so far yielded disappointing results in clinical trials [13], possibly because of its short plasma half-life and poor blood-brain barrier (BBB) penetration [14].

7,8-Dihydroxyflavone (7,8-DHF), a flavone derivative, has recently been identified as a specific TrkB agonist which passes the BBB after peripheral administration [15]. 7,8-DHF binds to the extracellular domain of TrkB, inducing its dimerization and autophosphorylation, and activates the downstream PI3K/Akt and Erk pathways in primary neurons [15], [16]. As a consequence of activating TrkB signaling, 7,8-DHF promotes survival and enhances neurite growth in cultured neurons [15], [16], and is neuroprotective in rodent models of ischemic stroke [15] and neurodegenerative diseases such as Alzheimer’s disease [17], [18], Parkinson’s disease [15] and Huntington’s disease [19]. This evidence indicates that 7,8-DHF, acting as a selective TrkB agonist, can be applied as a tool to investigate the role of BDNF/TrkB signaling. Nevertheless, it has not yet been determined whether this compound can protect against TBI.

Our aim in the present study is to determine whether activation of TrkB signaling by 7,8-DHF is protective against TBI in a mouse model of TBI and in an *in vitro* neuronal stretch model and to examine whether 7,8-DHF could promote the TrkB downstream PI3K/Akt or Erk pathways, and increase endogenous BDNF expression.

## Material and Methods

### Animals

All study protocols were approved by the Institutional Animal care and Use Committee at Cheng Hsin General Hospital(Animal permit number CHIACUC 102-02), and all animals were treated in accordance with the Guide for the Care and Use of Laboratory Animals published by the US National Institutes of Health (NIH Publication No. 85–23, revised 1996). For all experiments, male C57BL/6J mice (age 8–10 weeks, weight 23–28 g) were used.

### Drugs and antibodies

7,8-DHF was purchased from TCI America (D1916, Portland, OR). LY294002 was purchased from Cell signaling Technology (Danvers, MA, USA). K252a was purchased from Santa Cruz Biotechnology (Santa Cruz, CA). Antibodies were purchased from different commercial sources (Table 1).

**Table 1:** Antibodies used in immunofluorescence and western blot.

Primary antibody	Commercial source	Catalog number	Species	Antibody type	Working concentration
Phospho-TrkB Tyr705	Abcam	ab52191	Rabbit	Polyclonal	WB 1∶2000; IF 1∶100
Total TrkB	Santa Cruz	sc-8316	Rabbit	Polyclonal	WB 1∶5000
Phospho-Akt Ser473	Cell signaling	9271	Rabbit	Polyclonal	WB 1∶1000
Phospho-Akt Thr308	Cell signaling	4056	Rabbit	Monoclonal	WB 1∶1000
Total Akt	Cell signaling	9272	Rabbit	Polyclonal	WB 1∶2000
Phospho-Erk p44/42	Cell signaling	9101	Rabbit	Polyclonal	WB 1∶1000
Total Erk 44/42	Cell signaling	9102	Rabbit	Polyclonal	WB 1∶2000
Phospho-CREB Ser133	Cell signaling	9198	Rabbit	Monoclonal	WB 1∶1000; IF 1∶100
Total CREB	Cell signaling	9197	Rabbit	Polyclonal	WB 1∶2000
Cleaved caspase-3	Cell signaling	9661	Rabbit	Polyclonal	WB 1∶1000
Bcl-2	Santa Cruz	sc-492	Rabbit	Polyclonal	WB 1∶2000
Bax	Santa Cruz	sc-493	Rabbit	Polyclonal	WB 1∶1000
Phospho-Bad Ser136	Cell signaling	4366	Rabbit	Monoclonal	WB 1∶1000
GFAP	Invitrogen	13-0300	Rat	Monoclonal	IF 1∶200
NeuN	Millipore	MAB377	Mouse	Monoclonal	IF 1∶500
F4/80	Serotec	MCA497GA	Rat	Monoclonal	IF 1∶100

### Experimental protocol

The animals were randomized to different treatment groups by using computer-generated random numbers. All measurements described below were also done in a blinded manner. Four studies were conducted. The first study was to determine the optimal dose of 7,8-DHF. Following controlled cortical impact injury (CCI), animals were randomized into 3 groups: 1) CCI + vehicle, 2) CCI +20 mg/kg 7,8-DHF (DHF20), 3) CCI +50 mg/kg 7,8-DHF (DHF50). 7,8-DHF dissolved in 60% DMSO (0.1 ml) or a corresponding volume of vehicle (60% DMSO) was administered intraperitoneally (ip) 10 min after injury and subsequently daily for 3 days (10 min, 24 h, 48 h, and 72 h), and behavior deficits (n = 13–14/group) and brain water content (n = 6/group) were evaluated as the main outcomes. The doses of 7,8-DHF were selected based on data from our pilot study (comparing 5, 10, 20, and 50 mg/kg; which found 20 mg/kg provided more protection against behavioral deficits; Figure S1). The 4-dose regimen was chosen because our previous study showed that apoptotic-related signals last for over 3 days after CCI [20]. The results of the first study showed that 20 mg/kg DHF reduced both behavior deficits and brain water content, but 50 mg/kg had no effect on behavior deficits. Therefore, a dose of 20 mg/kg was chosen for all subsequent histology and biochemical experiments.

The second study was to evaluate the effect of DHF20 on histological damage, apoptosis, and TrkB-related signals. Animals were randomized into 3 groups (sham injury, CCI + vehicle, CCI + DHF20). Testing after injury was as follows: 1) histology at day 4 or 28 (n = 6/group), 2) western blot analysis at 1 h, day 1 or 4 (n = 5–9/group), 3) enzyme-linked immunosorbent assay (ELISA) at day 4 (n = 6–9/group) and 4) RT-PCR analysis at day 1 or 4 (n = 6–8/group).

The third study was to determine the role of PI3K/Akt in 7,8-DHF effects by using the specific PI3K inhibitor (LY294002) or the role of TrkB using a Trk antagonist (K252a). Animals were randomized into 5 groups. Mice received either: 1) intracerebroventricular (icv) pretreatment with saline (vehicle control for inhibitors) followed by DHF20 after CCI (S+ DHF20 group); 2) & 3) icv pretreatment with LY294002 or K252a followed by 60% DMSO (vehicle control for DHF20) after CCI; or 4) & 5) icv pretreatment with LY294002 or K252a followed by DHF20 after CCI (L+ DHF20 or K+ DHF20 group). TBI-induced brain lesion volumes were assessed at day 4 using cresyl violet histology (n = 5–6/group).

The fourth study was to investigate the therapeutic potential of delayed administration of 7,8 DHF after TBI. DHF20 or vehicle was delivered ip at 3 h post-injury, and the protective effect on brain tissue damage was examined using cresyl violet histology at day 4 (n = 4–5/group).

### Controlled cortical impact injury

The CCI model was used to induce TBI as previously described [21]. Briefly, mice were anesthetized with ip injection of sodium pentobarbital (65 mg/kg; Rhone Merieux, Harlow, UK) and a dental trephine drill was used to make a 5-mm craniotomy over the right parietal cortex, centered on the bregma, and 0.1 mm lateral to the midline. Injury was produced using a pneumatic piston with a 2.5 mm-rounded metal tip (4 m/sec velocity, 2 mm deformation depth). Mice were maintained at 37.0±0.5°C body temperature using a heated pad throughout the surgery and recovery period. Sham-operated animals underwent the same procedure as injured mice, except for CCI.

### Intracerebroventricular injection

LY294002 (200 nM in 0.5 µl of 0.9% saline), K252a (100 µM in 0.5 µl of 0.9% saline), or equal volume of saline was icv injected at 30 min before CCI as previously described [20]. Briefly, a 30-gauge needle of a Hamilton syringe was inserted into the lateral ventricle (0.5 mm posterior to the bregma, 1 mm right lateral to the midline, and 2 mm in depth). Then LY294002, K252a, or saline was infused with an infusion pump at a rate of 0.05 µl/min for 10 min. The needle was removed 20 min after the infusion to prevent reflux, and the CCI procedure was performed immediately thereafter.

### Metabolic characteristics assessment

Mice were anesthetized with an overdose of sodium pentobarbital (200 mg/kg, ip), and right atrial puncture was performed to collect venous blood. The collected blood was centrifuged (3500 g for 5 min), and the serum was stored on ice until analysis. Serum blood urea nitrogen (BUN), creatinine (CRE), and alanine aminotransferase (ALT) were measured by a chemistry autoanalyzer (Synchron Clinical System LX20; Beckman Coulter, Fullerton, CA) to assess renal and liver functions.

### Behavioral testing

Behavioral testing was performed before and at 1, 4, 7, 14, 21, and 28 days post-CCI. Animals were pre-trained for 3 days for both the rotarod and beam walking tests.

#### mNSS

The mNSS included motor, sensory, reflex, and balance tests [22]. One point was given for the inability to perform each test or for absence of a testing reflex, and neurological function was graded on a scale of 0–18.

#### Rotarod test

An accelerating rotarod was used to measure motor function and balance [23]. Briefly, the rotarod speed was slowly increased from 6 rpm to 42 rpm within 7 min and the time for mice to fall off was recorded.

#### Beam walking test

The test was used to assess fine motor coordination and function by measuring the ability of the animals to traverse an elevated beam [23]. The time for the mouse to traverse the beam (not to exceed 60 s) and the hindlimb performance as it crossed the beam (based on a 1 to 7 rating scale) were recorded [24]. A score of 7 was given when animals traversed the beam with two or less footslips; 6 was given when animals traversed the beam with less than 50% footslips; 5 was given for more than 50% but less than 100% footslips; 4 was given for 100% footslips; 3 was given for traversal with the affected limb extended and not reaching the surface of the beam; 2 was given when the animal was able to balance on the beam but not traverse it; 1 was given when the animal could not balance on the beam. For the rotarod and beam walking tests, three measurements per trial were recorded 1 h before CCI (baseline) and at 1, 4, 7, 14, 21, and 28 days post-CCI.

### Brain water content

Brain water content was used as a measure of brain edema, which occurred because of BBB breakdown post-injury. After decapitation (under anesthesia) at day 4 post-injury, the ipsilateral and contralateral cortex (in a 4-mm coronal section, 2 mm from the frontal pole) and the cerebellum (as internal control) were weighed (wet weight), baked at 100°C for 24 h, and reweighed (dry weight). Water content was determined as [(wet weight-dry weight)/wet weight] ×100% [25].

### Histology and immunohistochemistry

Following anesthesia, mice were transcardially perfused at day 4 (for Fluoro-Jade B [FJB] staining, terminal deoxynucleotidyl transferase-mediated dUTP-biotin nick end labeling [TUNEL], immunofluorescence, or cresyl violet histology) or day 28 (for cresyl violet histology) post-injury. Brains were removed, post-fixed in 4% paraformaldehyde overnight, cryoprotected with 30% sucrose containing 0.1% sodium azide (Sigma-Aldrich, St. Louis, MO, USA), and then sectioned coronally (10 µm) from the level of the olfactory bulbs to the visual cortex.

Frozen sections (10 µm) were stained with FJB (Chemicon, Temecula, CA), a marker of degenerating neurons, and TUNEL (In situ Cell Death Detection Kit, Roche Molecular Biochemicals, Mannheim, Germany) as previously described [20]. Double immunofluorescence was performed by simultaneous incubation of primary antibodies (anti-phospho-TrkB, or anti-phospho-CREB) with anti-neuronal nuclei antigen (NeuN, neuronal marker), anti-glial fibrillary acidic protein (GFAP, astrocyte marker), or anti-F4/80 (microglia marker) [20]. Antibody information is listed in Table 1.

### Contusion volume and hemispheric enlargement assessment

Contusion volumes, residual cerebral tissue ratios, and hemispheric enlargement ratios were quantified using cresyl violet-stained sections at 20 rostral-caudal levels that were spaced 200 µm apart as previously described [20]. Sections were analyzed using software ImageJ vision 1.46 (ImageJ, National Institutes of Health, Bethesda, MD). The volume measurement was computed by summation of the areas multiplied by the interslice distance (200 mm). The preservation of cerebral tissue was evaluated by the ratio of the volume of the ipsilateral remaining cerebral hemisphere to the volume of the corresponding contralateral cerebral hemisphere. Brain edema was assessed by calculating the percentage of hemispheric enlargement using the following formula: ([ipsilateral hemisphere volume – contralateral hemisphere volume]/contralateral hemisphere volume) ×100% [25]. Analysis was performed by two experimenters who were blinded to all animal groups. Inter-rater reliability was within 10%.

### Quantification of FJB and TUNEL staining

FJB and TUNEL staining was quantified on three consecutive sections from the injury core at the level 0.74 mm from the bregma. FJB- and TUNEL-positive cells were counted at a magnification of 200 in 3 randomly selected, non-overlapping fields with an area of 920×860 µm^2^. FJB-positive cells were expressed as cells/field. Quantification of TUNEL staining was expressed as (TUNEL-stained nuclei/DAPI-stained nuclei) ×100%. Analysis was performed by two experimenters who were blinded to all animal groups. Inter-rater reliability was within 10%.

### Western blot

Western blot was performed as previously described [20]. A 4-mm coronal section from the injured area over the right parietal cortex was collected at 1 h, 1 day, and 4 days following CCI or sham surgery. Cortical neuron cultures were collected at 24 h post stretch injury. All samples were centrifuged at 14,000 g for 30 min, and supernatants were used for further protein analysis. Protein concentration was determined by Bradford reagent at 595 nm. Protein samples were denatured in gel-loading buffer at 100°C for 5 min, separated by electrophoresis on sodium dodecyl sulfate-polyacrylamide gels, and transferred to Immobilon-P membranes (Millipore, Billerica, MA, USA). Membranes were blocked with 5% milk in PBS-XT and probed with primary antibodies. Antibodies used are listed in Table 1.

### Enzyme-linked immunosorbent assay

A 4-mm coronal section was taken from the injured area or the corresponding contralateral site over the parietal cortex of injured or sham animals at day 4 post-injury. BDNF was measured in brain homogenates using a commercially available ELISA kit (Abnova, Walnut, CA).

### Real–time quantitative reverse transcriptase polymerase chain reaction (RT-PCR)

A 4-mm coronal section was either taken from the ipsilateral or the contralateral cortices of injured or sham animals at days 1 and 4 post-injury. After extraction with the use of RNeasy Mini Kits (QIAGEN, Valencia, CA), RNA samples were subjected to reverse transcription with SuperScript II RNase H reverse transcriptase (Invitrogen, Carlsbad, CA). Realtime quantitative RT-PCR analysis was performed with an ABI PRISM 7900 sequence detector (Applied Biosystems, Foster City, CA). Thermal cycling was initiated with a 2-min incubation at 50°C, followed by a 10-min denaturation step at 95°C and 40 cycles at 95°C for 15 s and 60°C for 1 min. The primers and probes for BDNF (TaqMan Gene Expression Assay ID 00432069-ml) and β-actin (Rn00607939_s1) were obtained from Applied Biosystems. Relative quantities of the BDNF and β-actin mRNA were calculated with the previously described comparative threshold cycle method [25].

### Primary cortical neuron cultures, neuron stretch injury, cell viability and cytotoxicity assessment

All culture medium supplies were from Invitrogen (Invitrogen, Carlsbad, CA, USA). Primary cortical neuron cultures were prepared from embryonic C57BL/6 mice at 15.5 days as previously described [25]. Cerebral cortices from 8 to 10 embryos were isolated and digested in 0.5 mg/ml papain, dissociated in Hibernate-A medium (containing B27 supplement), and cultured on flexible membranes coated with poly-L-lysine in 6-well plates (Flexcell International Corporation, Hillsborough, NC, USA) at a density of 5×10^4^ cells/cm^2^. Cultures were maintained in neurobasal medium supplemented with B27, 10 units/ml penicillin, 10 mg/ml streptomycin, and 0.5 mg/ml glutamine. Three days after plating, arabinofuranosyl cytidine was added to inhibit proliferation of glial cells, and half of the medium was removed and replaced with fresh medium at 4 days. The cells were incubated at 37°C in an atmosphere containing 10% O_2_, 85% N_2_, and 5% CO_2_ and the cortical neurons were used at day 10 *in vitro*. The purity of neurons was >95%, as determined by counting microtubule-associated protein 2-positive cells.

Cortical neurons were stretched by rapid deformation of the silastic culture plates (Flexplates) using the Cell Injury Controller II (Custom Design and Fabrication; Virginia Commonwealth University, Richmond, VA) as previously described [20]. This device uses a regulated flow of compressed gas to rapidly pressurize cells cultured on flexible substrates, causing a radial stretch injury. In brief, the injury controller delivered one 50-ms pulse (28 psi) of compressed nitrogen, which resulted in a 10.2 psi peak pressure to the well and deformed the membrane 6.5 mm. The neurons were rapidly stretched 135% of their original length and were treated with 0.5 µM, 5 µM, or 10 µM of 7,8-DHF, respectively, immediately post-injury.

Cell viability and cytotoxicity were assessed 24 h post-injury using the 3-[4,5-dimethyl-2-thiazolyl]-2,5-diphenyl-2-tetrazolium bromide (MTT) reduction assay (Sigma-Aldrich; St. Louis, MO) and lactate dehydrogenase (LDH) release assay (LDH assay kit; Roche), respectively [20], [25]. Cells were incubated at 37°C for 2 h with MTT (0.5 mg/ml; Sigma-Aldrich), and then a solution of anhydrous isopropanol, HCl (0.1 N), and 0.1% Triton X-100 was added to dissolve the water-insoluble formazan. The optical density was determined at 570 nm. Cell viability was expressed as a percentage of the control culture. LDH release was used to quantify cytotoxicity in cultured neurons. Culture supernatants were collected, incubated with substrate mixtures, allowed to undergo a coupled enzymatic reaction that results in the conversion of iodonitrotetrazolium to formazan, and assessed spectrophotometrically for LDH activity at 500 nm. Percent cytotoxicity was calculated by subtracting LDH content in injured cells from total LDH in undamaged controls.

The experiments were repeated 4 times with different batches of primary cultures.

### Statistical analyses

Data are presented as the mean and standard error of the mean (mean ± SEM). One-way or two-way analysis of variance (ANOVA) followed by post-hoc Bonferroni evaluation was used for multiple groups to determine significant differences. Student's *t*-test was used to test the difference between two groups. Statistical significance was set at *P*<0.05.

## Results

### 7,8-DHF improved long-term neurobehavioral functions and reduced brain edema after TBI

**Figure 1:**
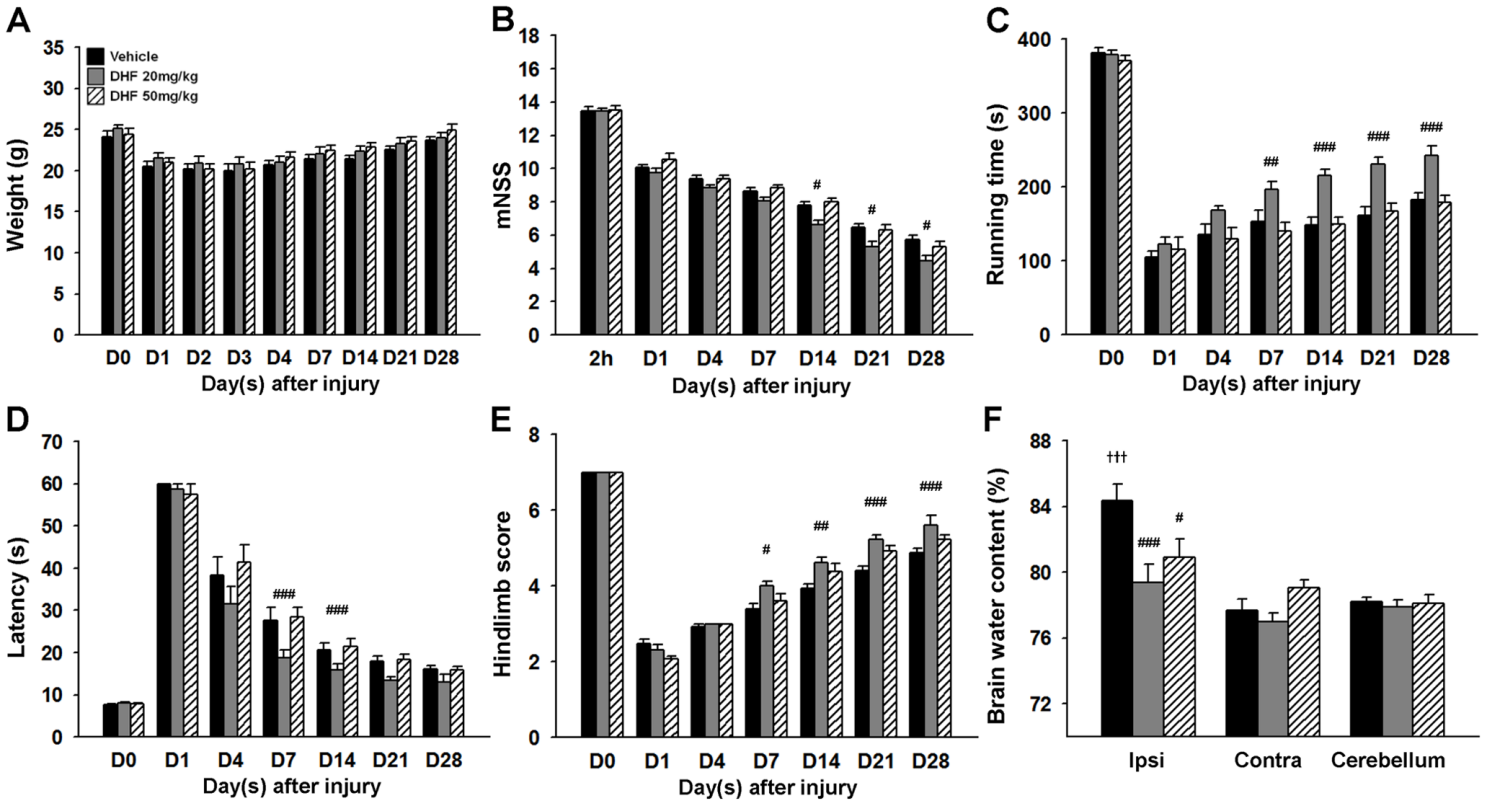
Post-injury 7,8-dihydroxyflavone treatment improved long-term neurobehavioral functions, reduced brain edema, but did not affect body weight change after TBI. **(A)** There was no significant differences in body weight change among the control (vehicle-treated), 20 mg/kg 7,8-dihydroxyflavone (DHF 20)-treated, and 50 mg/kg 7,8-dihydroxyflavone (DHF 50)-treated groups during the 28-day observation period post-TBI. DHF 20 (compared with vehicle) significantly **(B)** reduced the modified neurological severity score (mNSS) at days 7, 14, and 28 (all *P*<0.05) post-injury, **(C)** improved the rotarod performance from 7 to 28 days post-injury, **(D)** reduced the beam walk traversing time at days 7 and 14, and **(E)** improved hindlimb function from 7 to 28 days. Values are mean ± SEM; ^#^ *P*<0.05, ^##^ *P*<0.01, and ^###^ *P*<0.001 versus vehicle group (n = 13 or 14 mice/group, repeated measures two-way ANOVA). **(F)** Both DHF 20 and DHF 50 significantly attenuated brain water content in the ipsilateral hemisphere at 4 days. Values are mean ± SEM; ^#^ *P*<0.05 and ^###^ *P*<0.001 versus vehicle group,^ +++^ *P*<0.001 versus cerebellum (n = 6 mice/group, one-way ANOVA). Ipsi: ipsilateral cortex; Contra: contralateral cortex.

We first assessed the safety of 7,8-DHF in mice at 4 days after CCI when one dose of vehicle, DHF 20, or DHF 50 was administered daily for 4 days. There were no significant among-group differences in serum levels of BUN, CRE, indicators of renal function and ALT, an indicator of hepatic function (Table 2). Similarly, no significant among-group differences were found in body weight change (Figure 1A). Since both DHF groups showed no signs of toxicity, both doses (20 mg/kg and 50 mg/kg) were used for subsequent studies.

**Table 2:** Metabolic characteristics of the sham control, mice treated with vehicle and 7,8-dihydroxyflavone (20 or 50 mg/kg).

	CCI Day 4				
	Sham (n = 6)	Vehicle (n = 6)	20 mg/kg DHF (n = 5)	50 mg/kg DHF (n = 4)	Reference range
**BUN (mg/dL)**	23.25±3.20	20.97±1.12	26.38±3.91	18.3±0.55	8–33
**CRE (mg/dL)**	0.17±0.05	0.23±0.06	0.18±0.02	0.08±0.01	0.2–0.9
**ALT (mg/dL)**	39.67±13.59	45.83±5.59	49.00±8.65	22.5±2.02	17–77

Values are expressed as means ± SEM. CCI: cortical impact injury; BUN: blood urea nitrogen; CRE: creatinine; ALT: alanine aminotransferase; DHF: 7,8-dihydroxyflavone. Reference ranges [49], [50].

To evaluate the neuroprotective effects of 7,8-DHF, we first examined the functional outcomes. The mNSS was employed to measure global neurological impairment. At 2 h after injury, there was no difference in mNSS among groups treated with vehicle, DHF20, and DHF50, indicating that injury severity was the same initially regardless of treatment (Figure 1B). Improvement in neurological function at 14, 21, and 28 days was significantly better in the DHF20 group than in the control group (all *P*<0.05; Figure 1B). Rotarod and beam walking tests were used to assess motor and coordination function impairments, which manifest in many TBI patients who fails to ambulate independently [26]. Rotarod performance was significantly improved by DHF20 (compared with vehicle) at 7 days post-injury (*P*<0.01) and up to 28 days (*P*<0.001) (Figure 1C). In the beam-walking test, DHF20-treated mice required significantly less time to cross the beam at 7 and 14 days post-injury (both *P*<0.001; Figure 1D). Significant differences in hindlimb score were also observed between the DHF20-treated and vehicle-treated groups from 7 to 28 days (all *P*<0.05; Figure 1E). However, none of these functional tests detected significant effects of 7,8-DHF at the 50 mg/kg dose (Figures 1B–E). Taken together, these findings show that DHF20 can reduce long-term neurobehavioral deficits following CCI.

Because brain edema is a major characteristic and an important prognostic factor of TBI [27], we further measured brain water content to determine edema. At 4 days post-injury, CCI induced an increase of brain water content in the ipsilateral hemisphere of the vehicle-treated group compared with the contralateral hemisphere (84.4±2.4% versus 77.7±1.7%; *P*<0.001; Figure 1F). Brain water content was significantly attenuated in the ipsilateral hemisphere treated with both DHF20 and DHF50 when compared with the vehicle-treated group (DHF20: 79.4±2.7% versus 84.4±2.4%; *P*<0.001; DHF50: 80.9±2.2% versus 84.4±2.4%; *P* = 0.036). Based on the results showing that DHF20 reduced both functional deficits and brain edema, this dosage was used in the following studies.

### 7,8-DHF reduced brain tissue loss and neuronal damage after TBI

**Figure 2:**
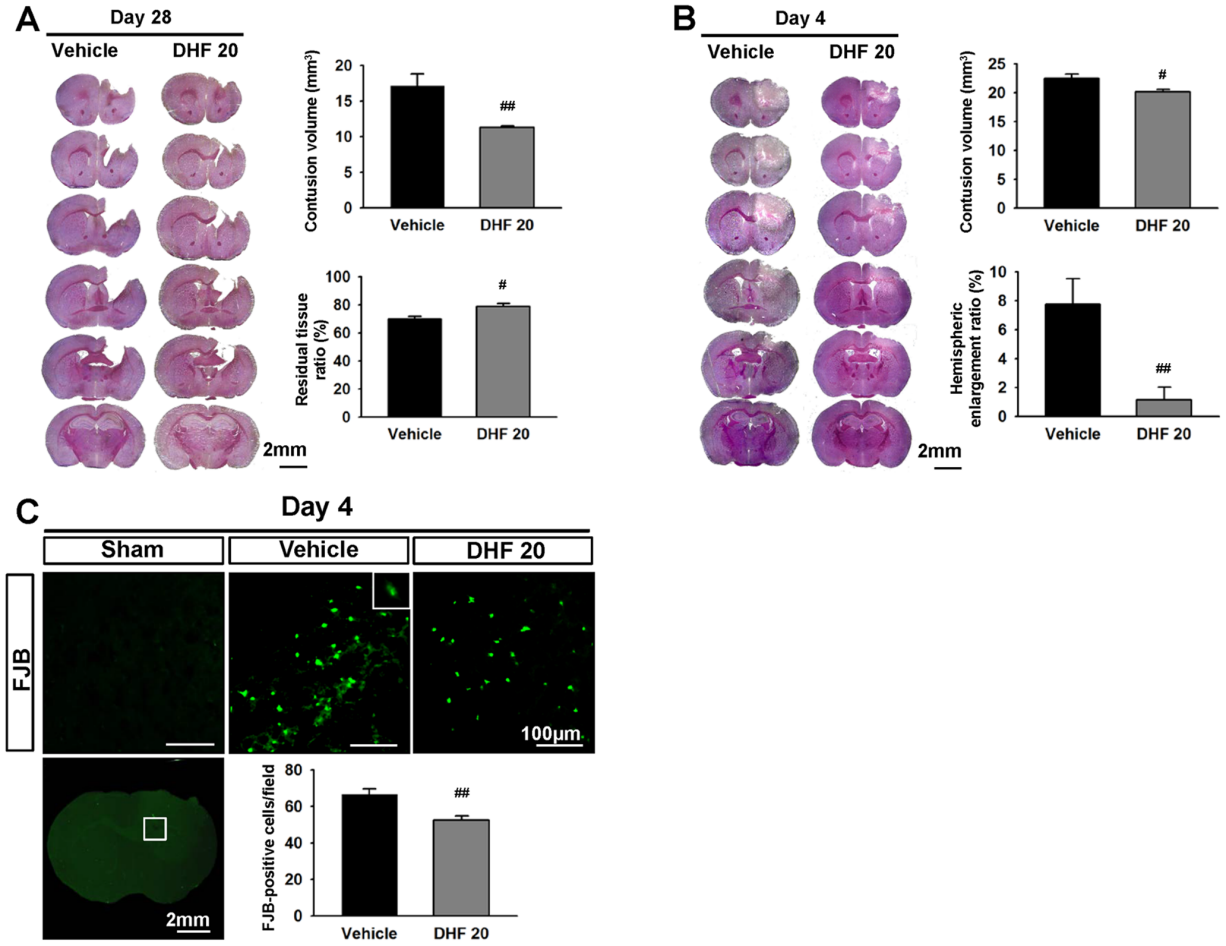
Post-injury 7,8-dihydroxyflavone treatment attenuated brain tissue and neuronal damage, and reduced hemispheric enlargement after TBI. Representative cresyl violet-stained brain sections of vehicle-treated and 20 mg/kg 7,8-dihydroxyflavone (DHF 20)-treated mice at **(A)** 4 and **(B)** 28 days post-TBI. Analysis of lesion volumes demonstrated that DHF 20 significantly reduced contusion volume and preserved brain tissue at 28 days and significantly reduced contusion volume and hemispheric enlargement at 4 days. The scale bar is 2 mm. **(C)** Representative Fluoro-Jade B (FJB)-stained sections of a sham-injured, vehicle-treated, and DHF 20-treated mouse at 4 days post-injury. The inset is a representative FJB-positive cell at higher magnification. Quantification analysis showed that DHF 20 mg significantly reduced the number of degenerating neurons at 4 days post-injury. The scale bar is 100 µm. Values are mean ± SEM: ^#^ *P*<0.05 and ^##^ *P*<0.01 versus vehicle group (n = 6 mice/group for both cresyl violet and FJB histochemistry, Student's *t*-test).

We next analyzed contusion volume and remaining ipsilateral hemisphere volume to see whether brain tissue preservation contributed to the long-term enhancement of functional recovery. DHF20 significantly reduced contusion volume to 66.1% of the vehicle-level from 17.1±4.2 mm^3^ to 11.3±0.6 mm^3^ at 28 days (*P* = 0.007; Figure 2A). We also measured remaining ipsilateral hemisphere volume because CCI induced tissue loss with atrophy of the ipsilateral cortex and striatum. Likewise, DHF20 significantly preserved brain tissue (78.3±5.2%) compared with vehicle treatment (69.7±5.5%, *P* = 0.015; Figure 2A), suggesting neuroprotection of the brain. Our group as well as other researchers previously reported that tissue loss during the chronic phase of TBI was associated with neurodegeneration at the acute stage [20], [28]. To evaluate whether DHF20 affects lesion volume and neurodegeneration at the acute stage, we further analyzed brain tissue damage and neuronal damage at 4 days post-injury. In parallel with the protective effect at 28 days, DHF20 significantly reduced contusion volume to 89.3% of the vehicle level from 22.5±1.8 mm^3^ to 20.1±1.1 mm^3^ at 4 days (*P* = 0.019; Figure 2B). We further measured the percentage of hemispheric enlargement to confirm the effect of DHF on brain water content. The percentage of hemisphere enlargement was significantly decreased in the DHF20-treated group compared with the vehicle-treated group (1.1±2.2% versus 7.8±4.3%; *P* = 0.007; Figure 2B). DHF20 also significantly reduced the number of FJB-positive neurons in the contusion margin of the injury core at 4 days (52.4±5.8 versus 66.7±7.2 cells/field, *P* = 0.004; Figure 2C). These results suggest that the enhancement of neurological recovery following DHF20 treatment may be due to increased neuronal viability and decreased brain tissue loss.

### 7,8-DHF reduced apoptosis after TBI

**Figure 3:**
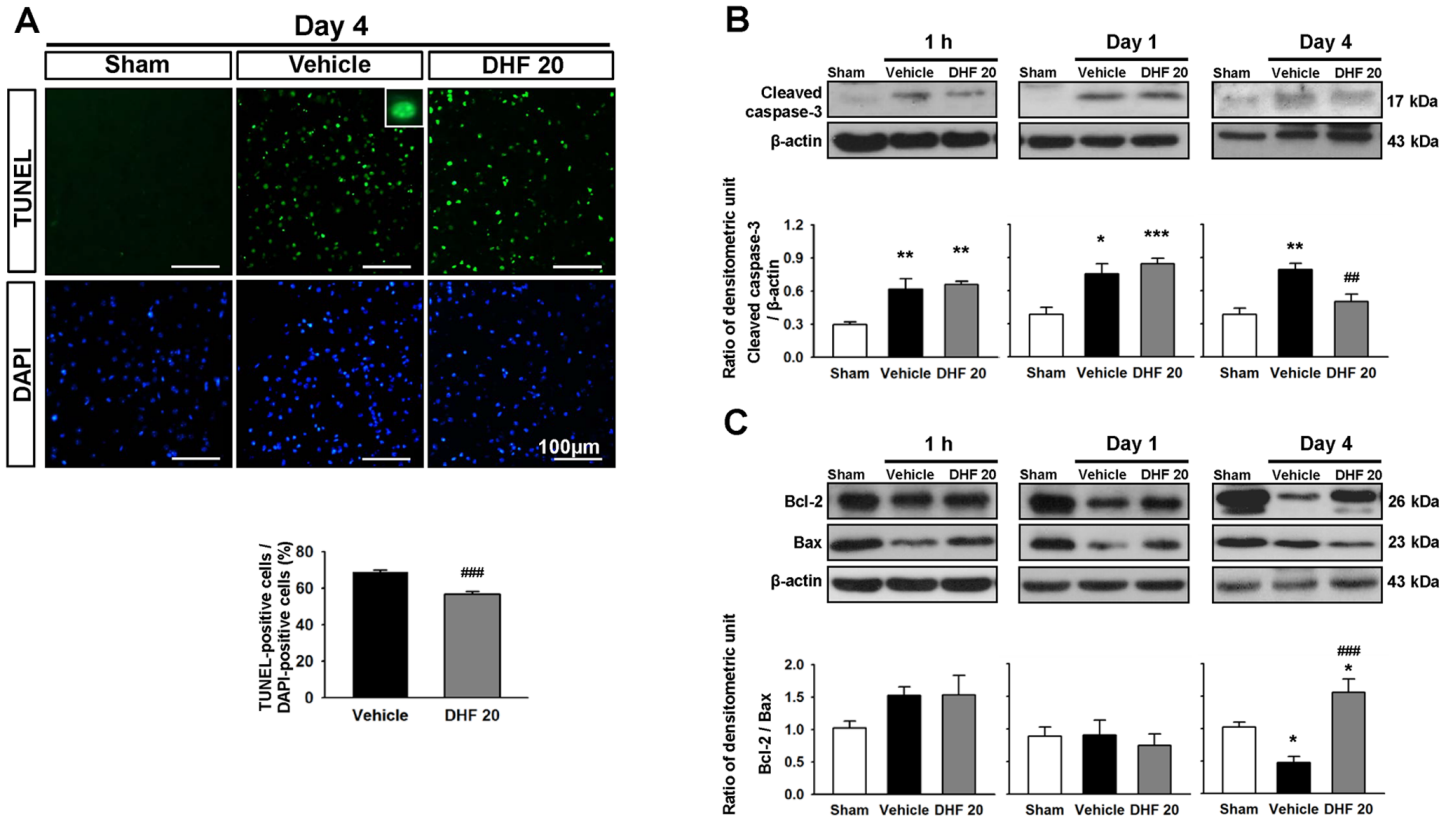
Post-injury 7,8-dihydroxyflavone treatment reduced apoptosis after TBI. (**A**) Representative terminal deoxynucleotidyl transferase-mediated dUTP-biotin nick end labeling (TUNEL, green) and DAPI-stained (blue) brain sections of a sham-injured, vehicle-treated, and 20 mg/kg 7,8-dihydroxyflavone (DHF 20)-treated mouse at 4 days post-TBI. The inset is a representative TUNEL-positive cell at higher magnification. Quantification showed that there were fewer TUNEL-positive cells in the DHF 20-treated group. The scale bar is 100 µm. Values are mean ± SEM; ^###^ *P*<0.001 versus vehicle group (n = 6 mice/group, Student's *t*-test). (**B**) Western blot analysis of apoptotic-related proteins (cleaved caspase-3, Bcl-2 and Bax) in the ipsilateral hemisphere of sham-injured, vehicle-treated, and DHF 20-treated mice at 1 h, 1 day, and 4 days post-CCI. DHF 20 mg/kg significantly decreased the cleaved caspase-3 level and increased the Bcl-2/Bax ratio at 4 days post-injury. Values are mean ± SEM; ^*^ *P*<0.05 and ^**^ *P*<0.01 versus sham group, ^##^ *P*<0.01 and ^###^ *P*<0.001 versus vehicle group (n = 5–8 mice/group, one-way ANOVA).

We then assessed the features of apoptosis to investigate whether DHF20-inducec brain tissue preservation reflects a change in cell apoptosis. Compared with vehicle, DHF 20 significantly diminished the number of TUNEL-positive cells in the contusion margin of the injury core at 4 days (56.8±3.1% versus 68.9±2.5%, *P*<0.001; Figure 3A). The level of cleaved caspase-3, a final effector of apoptotic death, was significantly elevated at all tested time-points (all *P*<0.05) in the vehicle-treated group (Figure 3B). At 4 days, the cleaved caspase-3 level in DHF-treated brains was significantly decreased to 64% (*P* = 0.019) of the vehicle-level (Figure 3B). We also measured the ratio between anti-apoptotic (Bcl-2) and pro-apoptotic (Bax) Bcl-2 family members because the imbalance of anti-apoptotic and pro-apoptotic Bcl-2 family proteins is an important cause of apoptotic cell death [3]. CCI caused a significant decrease in the Bcl-2/Bax ratio compared with sham-injury (47%, *P* = 0.04) at 4 days; however, DHF20 significantly raised the ratio to 325% (*P*<0.001) of the vehicle-level (Figure 3C). These results imply that 7,8-DHF may exert its protective effects by inhibiting progression of apoptosis after TBI.

### 7,8-DHF induced activation of TrkB and downstream PI3K/Akt signaling, but did not affect Erk signaling after TBI

**Figure 4:**
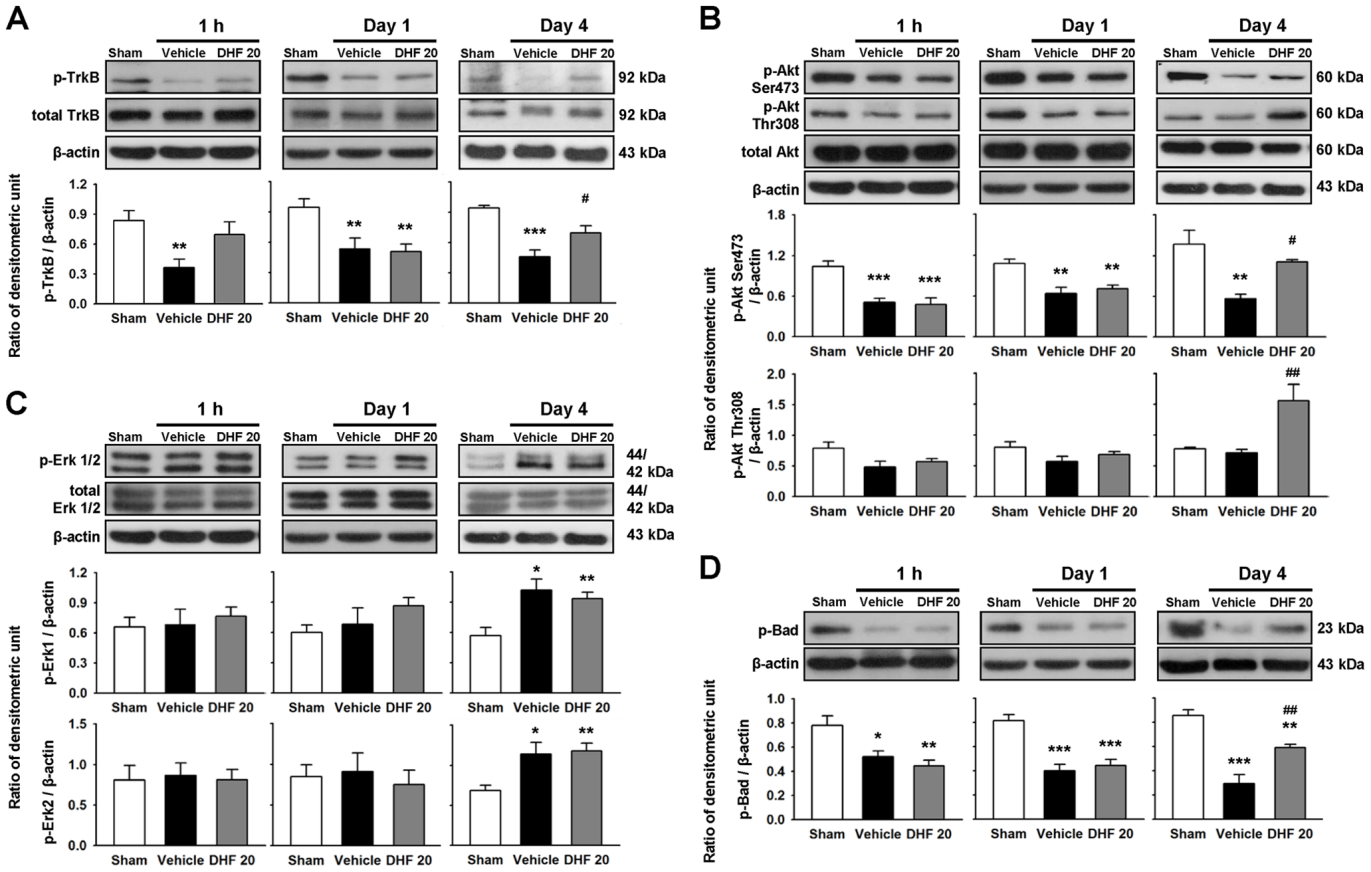
Post-injury 7,8-dihydroxyflavone treatment induced activation of TrkB and downstream PI3K/Akt signaling, but did not affect Erk signaling after TBI. Western blot analysis of **(A)** phospho-TrkB, **(B)** phospho-Akt, **(C)** phospho-Erk 1/2, and **(D)** phospho-Bad in the ipsilateral hemisphere of sham-injured, vehicle-treated, and 20 mg/kg 7,8-dihydroxyflavone (DHF 20)-treated mice at 1 h, 1 day, and 4 days post-TBI. DHF 20 significantly enhanced phosphorylation of TrkB, Akt at Ser473, Akt at Thr308, and Bad (the downstream factor of Akt) at 4 days post-injury. Erk 1/2 phosphorylation was not affected by DHF 20 at any tested time-points. Values are mean ± SEM; ^*^ *P*<0.05, ^**^ *P*<0.01, and ^***^ *P*<0.001 versus sham group, ^#^ *P*<0.05 and ^##^ *P*<0.01 versus vehicle group (n = 5–8 mice/group, one-way ANOVA).

To test whether DHF20 acted through TrkB, we examined phosphorylation of the TrkB and activation of its major downstream survival signaling pathways (the PI3K/Akt and Erk pathways). CCI induced a significant decrease of TrkB phosphorylation level at all tested time-points compared with sham-injury (all *P*<0.01). DHF20 significantly increased TrkB phosphorylation to 151% of its vehicle-level at 4 days (*P* = 0.04; Figure 4A). A similar trend was observed for Akt activation. The level of Akt Ser473 phosphorylation was significantly diminished following TBI at all tested time-points (all *P*<0.01) whereas the level of Akt Thr308 phosphorylation remained unchanged (all *P*>0.05; Figure 4B). The levels of both Akt phosphorylation forms were significantly higher in the DHF20 group than in the vehicle group at 4 days (phospho-Akt Ser473: 198% of vehicle, *P* = 0.032; phospho-Akt Thr308: 220% of vehicle, *P* = 0.004; Figure 4B) though differences at either 1 h or 1 day were insignificant. The level of Bad phosphorylation, a downstream factor of Akt, followed a very similar trend to that seen with the Akt phosphorylation. The Bad phosphorylation level significantly increased following DHF20 treatment (201% of vehicle, *P* = 0.018; Figure 4D) at 4 days post-injury. However, we did not detect any difference in the levels of Erk1/2 proteins following DHF treatment at all tested time-points (all *P*>0.05), although the Erk1/2 phosphorylation levels were increased in both injured groups at 4 days post-injury (Figure 4C). These findings demonstrate that 7,8-DHF provoked TrkB and downstream PI3K/Akt activation.

### 7,8-DHF increased brain BDNF levels and promoted CREB activation

**Figure 5:**
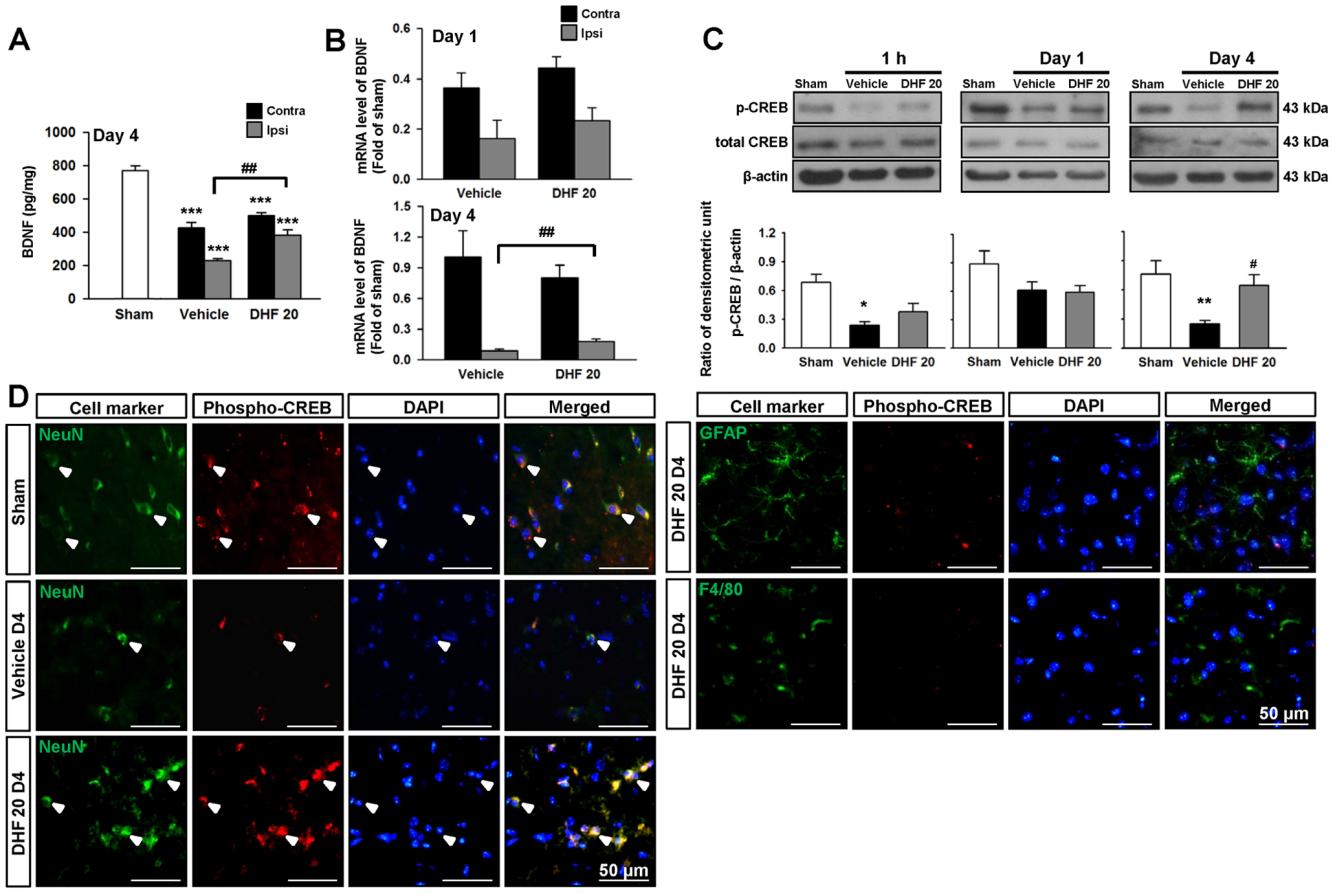
Post-injury 7,8-dihydroxyflavone treatment increased brain BDNF levels and promoted CREB activation. (**A**) Bar graphs demonstrating brain derived neurotrophic factor (BDNF) protein concentrations measured by enzyme-linked immunosorbent assay (ELISA) in sham-injured, vehicle-treated, and 20 mg/kg 7,8-dihydroxyflavone (DHF 20)-treated mice at 4 days post-TBI. DHF 20 significantly increased BDNF protein levels in the ipsilateral hemisphere at 4 days post-injury. (**B**) Bar graphs demonstrating BDNF mRNA expression measured by real–time quantitative reverse transcriptase polymerase chain reaction (RT-PCR) at 1 and 4 days post-injury. DHF 20 significantly increased BDNF mRNA levels in the ipsilateral hemisphere at 4 days post-injury. (**C**) Western blot analysis of the phospho-CREB level in the ipsilateral hemisphere of sham-injured, vehicle-treated, and DHF 20-treated mice at 1 h, 1 day, and 4 days post-injury. DHF 20 enhanced CREB phosphorylation at 4 days post-injury. (**D**) Identification of phospho-CREB-positive cells 4 day post-injury in the peri-contusional margin by double immunofluorescence staining. Phospho-CREB is shown in red, and NeuN (neurons), GFAP (astrocytes), or F4/80 (microglia) is shown in green. Co-localization of phospho-CREB with NeuN is shown by yellow labeling. Sections were stained with DAPI (blue) to show all nuclei. Values are mean ± SEM; ^*^ *P*<0.05, ^**^ *P*<0.01, and ^***^ *P*<0.001 versus sham group, ^#^ *P*<0.05, ^##^ *P*<0.01, and ^###^ *P*<0.001 versus vehicle group (n = 6–9 mice/group for BDNF protein and 7–9 mice/group for phospho-CREB, one-way ANOVA; n = 6–8 mice/group for BDNF mRNA, one-way ANOVA or Student's *t*-test). The scale bar is 50 µm. Ipsi: ipsilateral cortex; Contra: contralateral cortex.

Previous studies have shown that BDNF/TrkB signaling can self-amplify BDNF actions through its TrkB and end-product, CREB [8], [29]. To determine whether 7,8-DHF would trigger further BDNF production, we measured the brain levels of BDNF and CREB phosphorylation, a key transcription factor for BNDF induction. The BDNF protein levels were significantly decreased in both the contralateral and ipsilateral cortex at 4 days after CCI compared with sham-injury, but DHF20 substantially increased BDNF to 166% of the vehicle-level in the ipsilateral cortex (*P* = 0.002; Figure 5A). Similarly, the mRNA level of BDNF also increased to 203% of the vehicle-level in the ipsilateral cortex at 4 days after DHF20 treatment (*P* = 0.002; Figure 5B). Consistent with this, CCI induced a decrease in CREB phosphorylation level whereas DHF20 caused an increase in CREB phosphorylation level (158% of the vehicle-level, *P* = 0.021; Figure 5C) at 4 days. Double immunofluorescence further showed that CREB phosphorylation was mainly localized in neurons. The colocalization of p-CREB and NeuN decreased in the peri-contusional margin of vehicle-treated mice compared with the sham control at 4 days. However, DHF20 treatment increased CREB phosphorylation in neurons (Figure 5D). These data suggest that increased endogenous BDNF protein contributes to the neuroprotective effects of 7,8-DHF.

### Inhibition of Trk phosphorylation and PI3K/Akt activation blocked 7,8-DHF-induced neuroprotection

**Figure 6:**
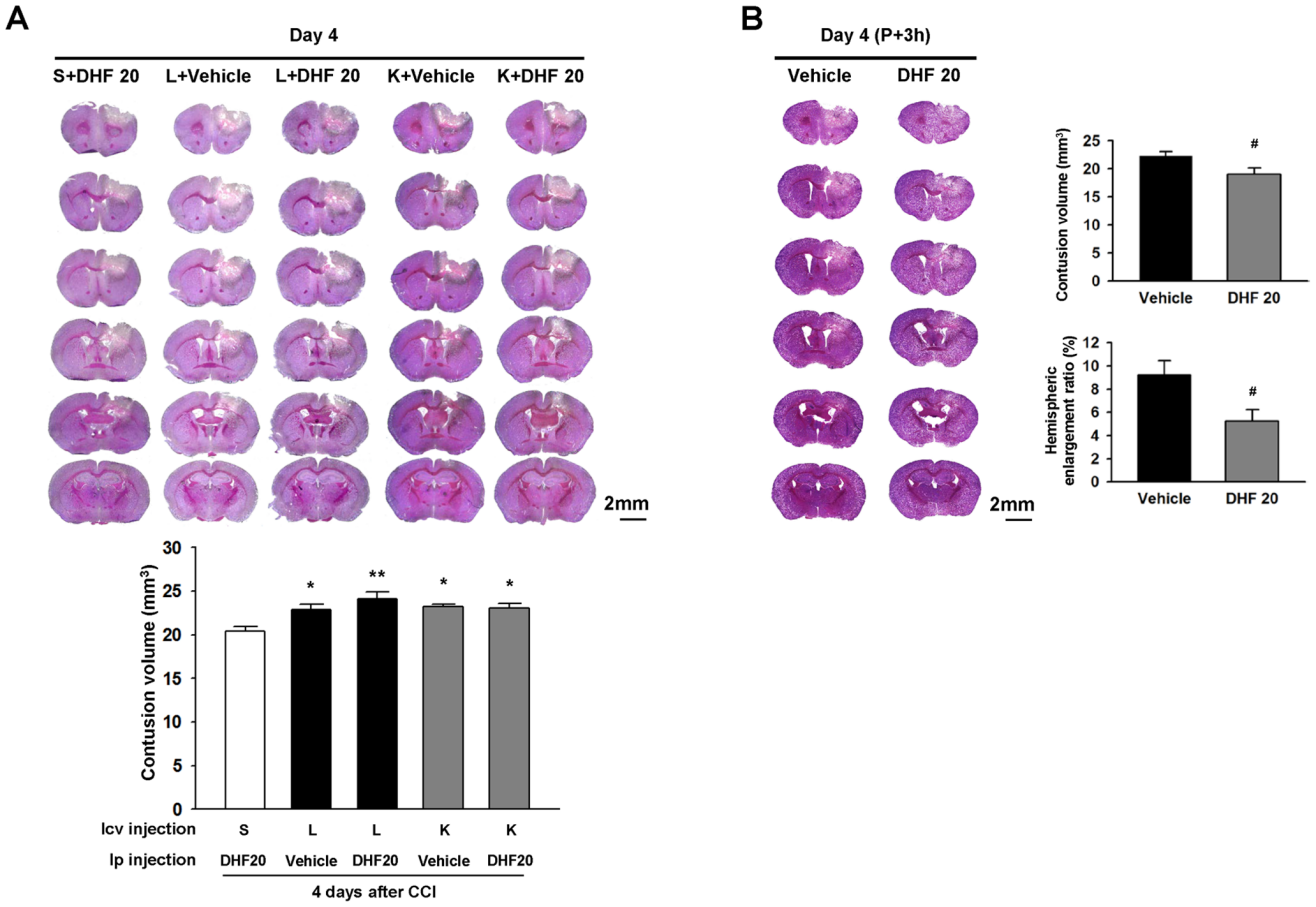
Inhibition of Trk phosphorylation or PI3K/Akt activation blocked 7,8-dihydroxyflavone-induced neuroprotection and delayed 7,8-dihydroxyflavone treatment protected against brain damage. **(A)** Representative cresyl-violet stained images from mice that received 1) intracerebroventricular (icv) pretreatment with saline followed by 20 mg/kg 7,8-dihydroxyflavone (DHF 20) after cortical impact injury (CCI) (S+ DHF 20), 2) either icv pretreatment with LY294002 (PI3K/Akt inhibitor) or K252a (TrkB inhibitor) followed by DMSO after CCI, or 3) either icv pretreatment with LY294002 (PI3K/Akt inhibitor) or K252a (TrkB inhibitor) followed by DHF 20 after CCI at 4 days post-injury. The scale bar is 2 mm. Quantification analysis showed that DHF 20-induced brain tissue protection was significantly attenuated in the LY294002 or K252a group. Values are mean ± SEM; ^*^ *P*<0.05 and ^**^ *P*<0.01 versus S + DHF 20 group (n = 5–6 mice/group, one-way ANOVA). S: Saline; L: LY294002; K: K252a. **(B)** Representative cresyl violet-stained brain sections of vehicle-treated and DHF-20-treated mice at 4 days post-TBI when administered at 3 h post-injury. Analysis of lesion volumes demonstrated that DHF 20 significantly reduced contusion volume and hemispheric enlargement at 4 days. The scale bar is 2 mm. Values are mean ± SEM; ^#^ *P*<0.05 versus vehicle group (n = 4–5 mice/group, Student's *t*-test).

To further confirm that the beneficial effect of 7,8-DHF is dependent on activation of TrkB and the PI3K/Akt pathway, the Trk receptor inhibitor K252a and a specific PI3K inhibitor LY294002 were administered to DHF20-treated CCI mice. Inhibiting of TrkB phosphorylation with K252a significantly abolished DHF20-induced reduction of contusion volume at 4 days post-injury. The injury volume of the K252a (K) + DHF20 group was increased up to 113% of the saline (S) + DHF20 group from 20.4±0.5 mm^3^ to 23.1±0.5 mm^3^ (*P* = 0.049; Figure 6A). Similarly, LY294002 significantly obliterated the protective effect of DHF20. The injury volume of the LY294002 (L) + DHF20 group was increased to 118% of the S + DHF20 group from 20.4±0.5 mm^3^ to 24.1±0.7 mm^3^ (*P* = 0.002; Figure 6A). However, the injury volumes of the L+ DHF20 and K+ DHF20 groups were similar (*P* = 0.302). These data indicate that 7,8-DHF-induced brain tissue protection in CCI brains is mediated through TrkB and downstream PI3K/Akt pathways, and that the respective protective effects of TrkB and PI3K/Akt are similar.

We next examined the protective effect of 7,8 DHF when treatment was initiated at 3 h post-injury. DHF20 significantly reduced contusion volume to 85% of the vehicle-level from 22.3±0.8 mm^3^ to 19.1±1.1 mm^3^ at 4 days (*P* = 0.048; Figure 6B), and the percentage of hemisphere enlargement was also significantly decreased (9.2±1.2% versus 5.2±1.0%; *P* = 0.046). The effect of treatment initiated at 10min or 3 h post-injury was similar (Figures 1H & 5B).

### Treatment with 7,8-DHF attenuated neuronal death in the in vitro TBI model

**Figure 7:**
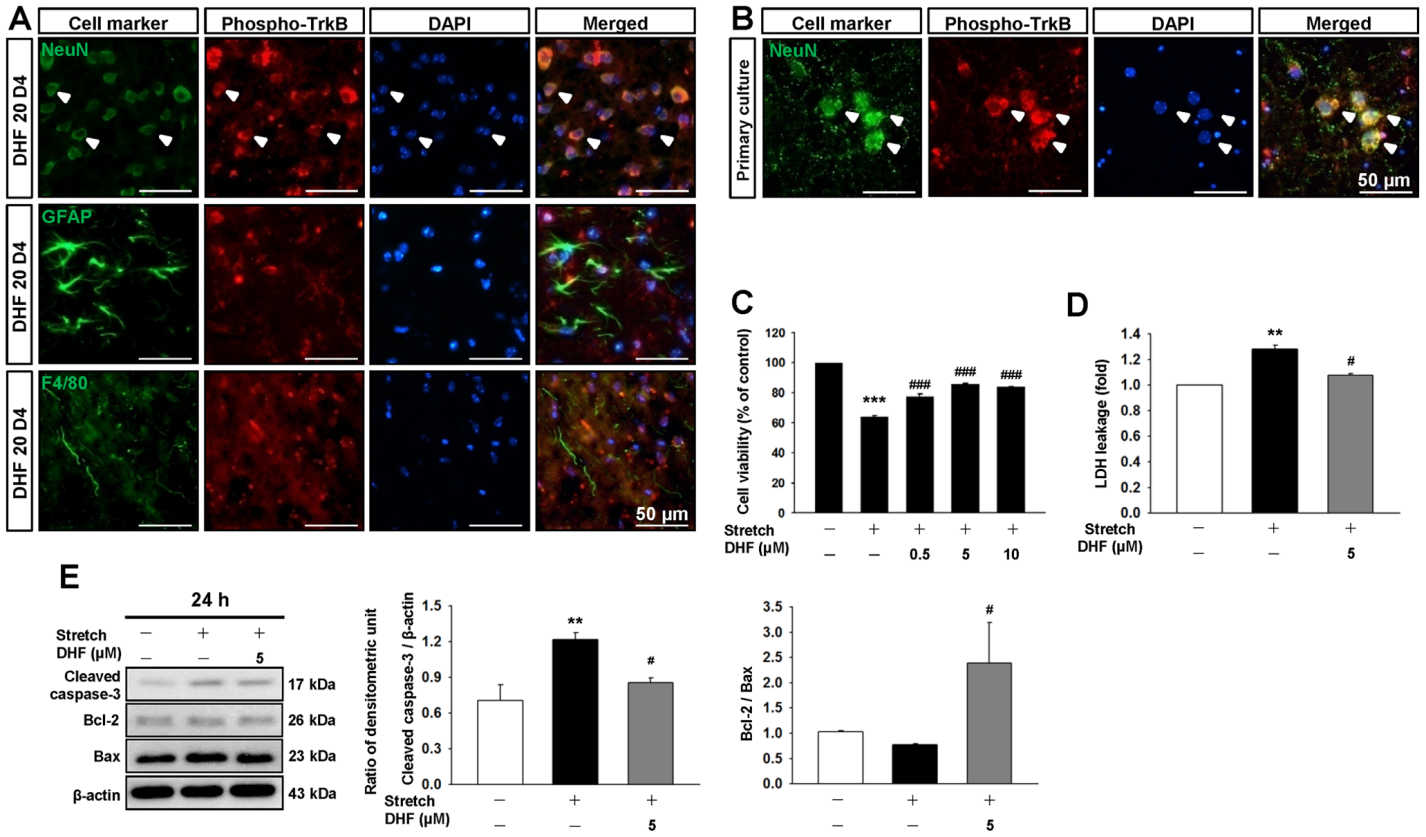
The TrkB was expressed in neurons in the injured brain and treatment with 7,8-dihydroxyflavone attenuated stretch-induced cytotoxicity and apoptosis in primary cultured neurons. **(A)** Cellular localization of phospho-TrkB is in the peri-contusion margin at 4 days post-TBI observed by immunofluorescence labeling. Phospho-TrkB is shown in red, and NeuN (neurons), GFAP (astrocytes), or F4/80 (microglia) is shown in green. Colocalization of phospho-TrkB with neurons but not with astrocytes or microglia is shown by yellow labeling. Sections were stained with DAPI (blue) to show all nuclei. The scale bar is 50 µm. **(B)** Colocalization of phospho-TrkB (red) with NeuN (green) by double immunofluorescence staining at 24 h post-stretch injury is indicated by yellow labeling (white arrows). The scale bar is 50 µm. **(C)** Treatment with 0.5 µM, 5 µM, or 10 µM 7,8-dihydroxyflavone (7,8-DHF) immediately after stretch injury increased cell viability by 3-[4,5-dimethyl-2-thiazolyl]- 2,5-diphenyl-2-tetrazolium bromide (MTT) assay, and **(D)** treatment 0f 5 µM 7,8-DHF reduced cytotoxicity in primary cultured neurons as assessed by lactate dehydrogenase (LDH) assay. **(E)** Western blot analysis of apoptosis-related proteins (cleaved caspase-3, Bcl-2, and Bax) shows that 5 µM DHF significantly decreased the cleaved caspase-3 level and the Bcl2/Bax ratio. Values are presented as mean ± SEM of 3 or 4 independent experiments (n = 3 or 4). ^**^ *P*<0.01 and ^***^ *P*<0.001 versus normal control, ^#^ *P*<0.05, ^###^ *P*<0.01, and ^###^ *P*<0.001 versus stretch injury alone.

To further investigate whether TrkB activation induced by 7,8-DHF directly occurred in neurons, we examined the localization of the phospho-TrkB following CCI. TrkB phosphorylation was found mainly in neurons around the impact site, but rarely in astrocytes or microglia (Figure 7A). We next assessed the impact of 7,8-DHF on damaged neurons in an *in vitro* stretch injury model. Using double immunofluorescence, we confirmed that TrkB phosphorylation occurred in primary cortical neurons (Figure 7B). Stretch injury of cortical neurons significantly induced loss (monitored by MTT assay) of neuron viability (61.8±1.5% of cell viability relative to control, *P*<0.001; Figure 7C). Treatment with 0.5 µM, 5 µM, or 10 µM 7,8-DHF immediately post-injury increased cell viability to 120%, 134%, and 131% of the control-level (all *P*<0.001) and 5 µM provided the most protection (Figure 7C). In parallel, 5 µM 7,8-DHF significantly reduced stretch-induced cytotoxicity from 28% of the normal control to 8% by LDH assay (*P*<0.001; Figure 7D). Therefore, the dosage of 5 µM was employed for subsequent biochemical studies. Finally, we assayed apoptosis-related molecules to determine whether the protective effect of 7,8-DHF on cortical neurons was also mediated through apoptosis inhibition. 7,8-DHF (5 µM) significantly prevented stretch-induced apoptosis in cortical neurons. The cleaved caspase-3 level in DHF-treated neurons was significantly decreased to 70% (*P* = 0.047) and the Bcl-2/Bax ratio was significantly increased to 309% (*P* = 0.048) of the control-level (Figure 7E).

## Discussion

This study shows for the first time activation of the TrkB by 7,8-DHF improved long-term functional recovery and attenuated brain tissue damage, brain edema, and apoptosis following experimental TBI. 7,8-DHF also promoted activation of the downstream PI3K/Akt pathway and enhanced BDNF expression and CREB activation. These *in vivo* findings correlated with the compound's ability to improve neuronal survival and reduce apoptosis in an *in vitro* stretch injury model. Although previous studies have shown that prophylactic or immediate post-injury 7,8-DHF treatment can reduce brain damage in experimental models of cerebral ischemia [15], [30], our study provides the first evidence that post-injury 7,8-DHF treatment is neuroprotective against TBI. Remarkably, 7,8 DHF also exerted significant protective effects using a more clinically relevant post-injury administration paradigm. Our results suggest that 7,8-DHF may provide a potential therapy for TBI.

Accumulating evidence has documented the critical role of TrkB signaling in promoting neuronal survival [7]. TrkB activation prevents neuronal degeneration in various *in vitro* models of neuronal insults [15], [31], [32]. TrkB activation also reduces functional and histological damage following experimental cerebral ischemia [15], [33]. Although activation of TrkB signaling by small molecule BDNF mimetics reversed motor deficits caused by rat TBI, it did not attenuate brain tissue damage and the precise mechanisms of the protective effect were not investigated [32]. We showed that TBI induced a reduction of TrkB and Akt activation. The ability of 7,8-DHF to preserve TrkB and its downstream Akt activation at the acute stage was paralleled by a reduction of functional and histological deficits over one month. Our results suggest that enhancing TrkB activation attenuates acute neuropathological events, which consequently leads to better neurological recovery over a long period.

We demonstrated that 7,8-DHF increased Akt phosphorylation at both Ser473 and Thr308 residues, without affecting Erk 1/2 phosphorylation following mouse TBI. Our results contradict previous *in vitro* findings that 7,8-DHF increased both Akt and Erk 1/2 phosphorylation in primary neurons [15]. This disparity may be attributable to variability in the types of insults and to difficulty in drawing parallels between *in vitro* and *in vivo* studies. The observed Erk activation in previous *in vitro* work occurred in cultured neurons not subjected to any toxic insults [15]. Indeed, previous studies have reported that the relative contribution of the PI3K/Akt and Erk pathways to BDNF neuroprotection depended on the types of cellular injury [7]. Furthermore, the contribution of the Erk pathway to neuronal survival is controversial as growing evidence suggests that Erk activation is prominently involved in neurodegeneration in TBI, ischemia, and Alzheimer's disease [34]. We showed that TBI induced a significant increase of Erk1/2 phosphorylation levels at 4 days post-injury while reducing TrkB and Akt Ser473 phosphorylation levels, suggesting that PI3K/Akt and Erk pathways may play different roles in mediating neuronal survival. Our data emphasize the importance of defining survival pathways that counteract apoptosis induced during brain injury. An alternative explanation for the controversial reports about involvement of the Erk pathway is the cell-type-specific response to 7,8-DHF treatment. For example, 7,8-DHF down-regulated the Erk pathways in the macrophage cell line [35].

We observed that 7,8-DHF increased Akt phosphorylation, coupled with an increase of Bad phosphorylation, the Akt downstream target. Blocking Akt activation using a specific PI3K inhibitor abolished 7,8-DHF-induced protection against brain tissue damage, indicating that DHF-induced neuroprotection was dependent on the PI3K/Akt signaling pathway. We and other researchers have demonstrated that the PI3K/Akt is a major survival pathway in TBI [20], [36]. Akt inhibits apoptosis by phosphorylating and inactivating pro-apoptotic factors (such as Bad) to maintain mitochondrial integrity through preventing the inhibition of anti-apoptotic Bcl-2 by Bad [1]. Consistent with increased phosphorylation of Akt and Bad, 7,8-DHF increased the Bcl-2/Bax ratio, a rheostat that regulates apoptosis, and reduced the cleaved caspase-3 level as well as TUNEL-positive cells following mouse TBI. In line with the *in vivo* findings, 7,8-DHF promoted neuronal survival, increased the Bcl-2/Bax ratio, and reduced apoptosis in the *in vitro* stretch injury model. Although the Bcl-2/Bax ratio in the stretch group was reduced to about 74% of the control-level in the *in vitro* stretch injury model, it did not reach statistical significance. Since the injury severity we used was moderate stretch, it is possible that the moderate degree of stretch does not induce a decrease of Bcl-2/Bax. Indeed, a previous study using the same stretch model reported that only severe stretch caused a decline in mitochondrial function at 24 h but moderate stretch didn't affect mitochondrial function until 48 h [37]. Another explanation is the time point examined. The Bcl-2/Bax ratio might change at other time points following stretch as the Bcl-2/Bax ratio remained unchanged at 1 h and 1 day following mouse TBI but decreased at day 4. Our data support the notion that TrkB-induced activation of PI3K/Akt plays an important role in rescuing neurons from traumatic insults. However, previous studies have suggested that 7,8-DHF can induce neuroprotection through its antioxidant action *in vitro* [38], [39]. The beneficial effects of 7,8-DHF may be attributed in part to the prevention of free radical and oxidant formation. Further studies are required to clarify the anti-oxidative mechanism underlying the 7,8-DHF-mediated neuroprotection in TBI.

A novel finding in this study was that both BDNF protein and mRNA in the injured cortex was increased after 7,8-DHF treatment following TBI. This observation could possibly be explained by a positive feedback mechanism, in which an initial enhancement of TrkB phosphorylation following 7,8-DHF treatment may activate the PI3K/Akt pathway, stimulating the synthesis of BDNF via CREB, an important transcription factor needed to regulate BDNF transcription [8]. The increased endogenous BDNF could subsequently bind to the TrkB protein and further amplify the TrkB/Akt signaling. We found that BDNF mRNA and protein expression was decreased at both the ipsilateral and contralateral cortex following cortical impact injury. Our results are different from previous studies showing that the mRNA expression of BDNF decreased in the ipsilateral hippocampus or cortex while there was an increase in mRNA expression at the contralateral side following fluid percussion injury or penetrating brain injury [40], [41]. This inconsistency may be due to differences in injury models, injury severity, regions of interest or time points examined. Evidence shows that CREB activation could be regulated by at least three pathways, including the Erk, PI3K/Akt, and cAMP-dependent signaling pathways [14]. We demonstrated that 7,8-DHF increased phosphorylation of CREB, Akt, and its downstream target Bad but not Erk, suggesting that the positive feedback loop of TrkB signaling in the injured brain is via the PI3K/Akt-CREB signal transduction cascade. The activation of Akt/PKB in neurons has also been linked at the transcriptional level to the phosphorylation of CREB [9]. Nevertheless, the mechanisms underlying CREB phosphorylation triggered by TrkB-induced Akt activation need further investigation.

We observed that 7,8-DHF 20 mg/kg but not 50 mg/kg improved functional recovery. Our results were in agreement with previous *in vitro* results showing that higher dosages of 7,8-DHF decreased the viability of cultured neurons [16], [38]. The reduced efficiency of the higher dose of 7,8-DHF (which had no effect on post-injury body weight loss, and renal or liver function in our study) may be attributed to the pharmacological profiles of flavonoids (which have different pharmacological effects depending on their concentration) [42]. For example, Akt activation in a hepatoma cell line is induced by lower concentrations of the flavonoid quercetin and inhibited by higher concentrations [43]. An alternate explanation is that 7,8-DHF, acting as a potent BDNF agonist, may have dose-dependent neuroprotective activity similar to that of BDNF [44]. Previous studies have reported that high concentrations of BDNF or other TrkB ligands resulted in a downregulation of the TrkB response [45] and a decrease in the BDNF-mediated neuroprotection [44]. Our findings underscore the importance of addressing the possibility of ligand-induced down-regulation of the receptor response when considering TrkB agonists for treatment of neurological diseases.

The dosage of 7,8-DHF (20 mg/kg) used in this study was higher relative to the one used in most previous studies with mouse models of neurodegenerative diseases [17]-[19] or stroke (5 mg/kg) [15]. One possible explanation for the dose discrepancy between our and previous studies is different treatment schedules. In our study, 7,8-DHF was administered ip 10 min or 3 h after injury and subsequently once daily for 3 days (10 min, 24 h, 48 h, and 72 h). On the other hand, 7,8-DHF was administered for longer periods in most previous studies with neurodegenerative animal models, ranging from 10 to 14 daily ip injections in Alzheimer's disease mice [17], [18] to multiple oral doses for up to 14 weeks in Huntington's disease mice [19] . Another reason could be a discrepancy between therapeutic windows. While previous studies in animal models of cerebral ischemia applied 7,8-DHF treatment 2 h before injury [15], [46], 7,8-DHF was administered at 10 mins or 3 h post-injury in our study. Clearly, a post-treatment paradigm and wider therapeutic window would be more clinically feasible as prophylactic treatment is inapplicable due to the unpredictability of TBI. The dose disparity may also be due to different pathology from disease to disease. Indeed, different types of primary insults may result in diverse cellular vulnerability patterns as well as a spectrum of injury processes [47]. For example, TBI involves a primary mechanical impact which triggers several damaging pathways that lead to secondary neuronal death [47]. However, in neurodegenerative diseases such as Alzheimer's disease and Huntington's disease, the aggregation of misfolded protein has been regarded as a major pathological event [48].

In conclusion, we demonstrated that activation of the TrkB by 7,8-DHF reduced apoptosis, ameliorated functional deficits and brain damage following TBI, and improved survival in cultured cortical neurons. Our findings suggest that pharmacological enhancement of TrkB signaling by 7,8-DHF could be a potential strategy for TBI.

## Supporting Information

Figure S1 **Effects of 4 different doses of 7,8-dihydroxyflavone in contusion-injured mice.** Treatment with 20 mg/kg 7,8-dihydroxyflavone (DHF 20) significantly (**A**) reduced the modified neurological severity score (mNSS) at day 14 post-injury, (**B**) improved the rotarod performance at days 7 and 14 post-injury, and (**C**) reduced the beam walk traversing time from 4 to 14 days. Values are mean ± SEM; ^#^ *P*<0.05, ^##^ *P*<0.01, and ^###^ *P*<0.001 versus vehicle group (n = 7–9 mice/group, repeated measures two-way ANOVA).
(TIFF)
