# Supplementary figures and images for: Post-Injury Treatment with 7,8-Dihydroxyflavone, a TrkB Receptor Agonist, Protects against Experimental Traumatic Brain Injury via PI3K/Akt Signaling
Source: PLoS One. 2014 Nov 21;9(11):e113397. doi: 10.1371/journal.pone.0113397 (PMC4240709; doi:10.1371/journal.pone.0113397)

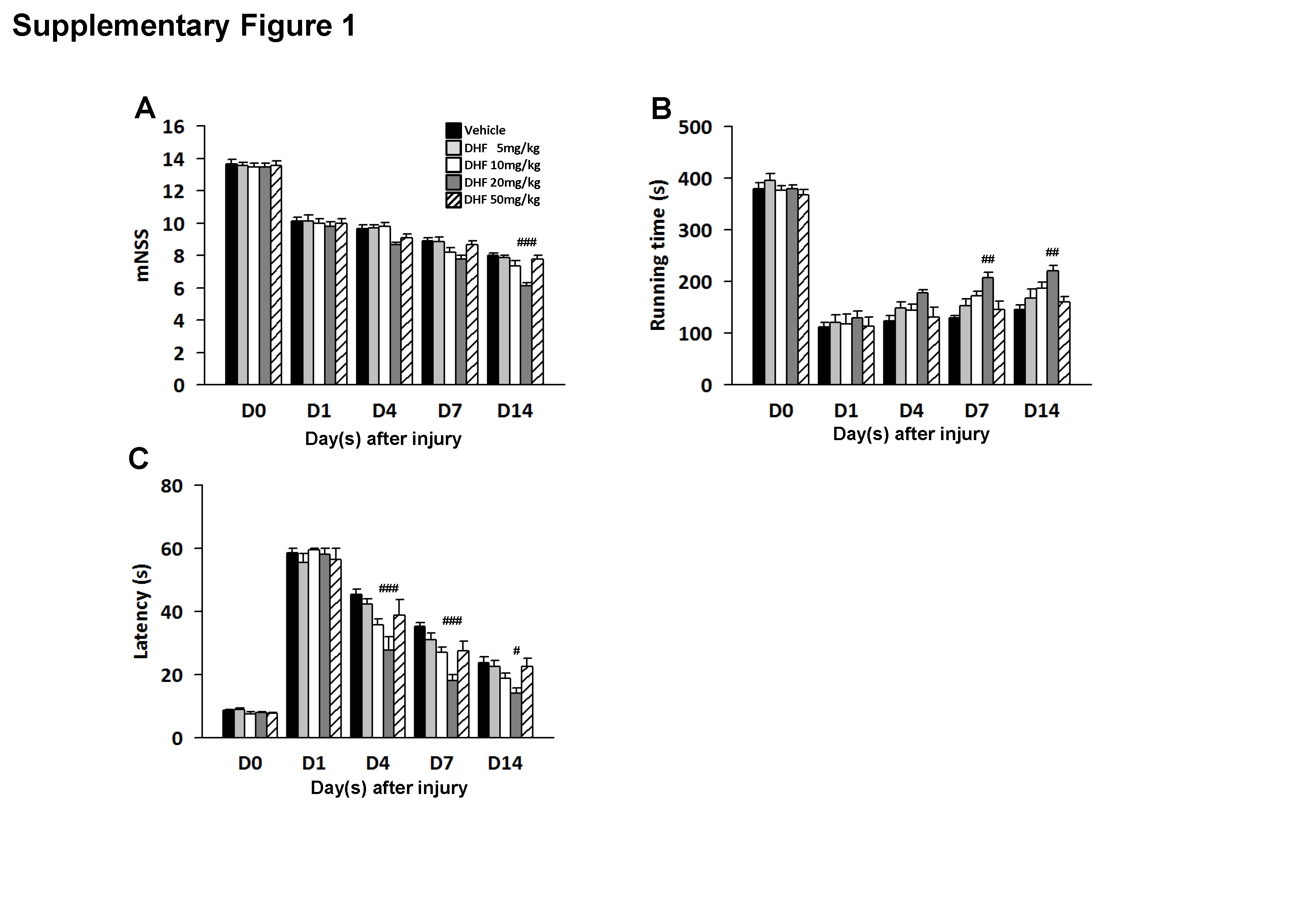

Supplement: Figure S1 — Effects of 4 different doses of 7,8-dihydroxyflavone in contusion-injured mice. Treatment with 20 mg/kg 7,8-dihydroxyflavone (DHF 20) significantly (A) reduced the modified neurological severity score (mNSS) at day 14 post-injury, (B) improved the rotarod performance at days 7 and 14 post-injury, and (C) reduced the beam walk traversing time from 4 to 14 days. Values are mean ± SEM; # P<0.05, ## P<0.01, and ### P<0.001 versus vehicle group (n = 7–9 mice/group, repeated measures two-way ANOVA). (TIFF) [file pone.0113397.s001.tiff]
